# Biodegradable and Antimicrobial PLA–OLA Blends Containing Chitosan-Mediated Silver Nanoparticles with Shape Memory Properties for Potential Medical Applications

**DOI:** 10.3390/nano10061065

**Published:** 2020-05-30

**Authors:** Agueda Sonseca, Salim Madani, Alexandra Muñoz-Bonilla, Marta Fernández-García, Laura Peponi, Adrián Leonés, Gema Rodríguez, Coro Echeverría, Daniel López

**Affiliations:** 1 MacroEng Group, Instituto de Ciencia y Tecnología de Polímeros, ICTP-CSIC, C/Juan de la Cierva 3, 28006 Madrid, Spain; sbonilla@ictp.csic.es (A.M.-B.); martafg@ictp.csic.es (M.F.-G.); lpeponi@ictp.csic.es (L.P.); aleones@ictp.csic.es (A.L.); gema@ictp.csic.es (G.R.); cecheverria@ictp.csic.es (C.E.); 2 Interdisciplinary Plataform for “Sustainable Plastics towards a Circular Economy” (SUSPLAST-CSIC), 28006 Madrid, Spain; 3 Laboratory of Applied Biochemistry, Department of Biology, Faculty of Sciences, University Ferhat Abbas, Setif 19000, Algeria; madanisalim79@gmail.com

**Keywords:** poly (lactic acid), oligomeric lactic acid, eco-friendly silver nanoparticles, shape memory properties, antimicrobial activity, biomedical

## Abstract

To use shape memory materials based on poly (lactic acid) (PLA) for medical applications is essential to tune their transition temperature (T_trans_) near to the human body temperature. In this study, the combination of lactic acid oligomer (OLA), acting as a plasticizer, together with chitosan-mediated silver nanoparticles (AgCH-NPs) to create PLA matrices is studied to obtain functional shape memory polymers for potential medical applications. PLA/OLA nanocomposites containing different amounts of AgCH-NPs were obtained and profusely characterized relating their structure with their antimicrobial and shape memory performances. Nanocomposites exhibited shape memory responses at the temperature of interest (near physiological one), as well as excellent shape memory responses, shorter recovery times and higher recovery ratios (over 100%) when compared to neat materials. Moreover, antibacterial activity tests confirmed biocidal activity; therefore, these functional polymer nanocomposites with shape memory, degradability and biocidal activity show great potential for soft actuation applications in the medical field.

## 1. Introduction

The use of shape memory polymers (SMP) for the development of active medical devices is perhaps the most promising and attractive area of application for these materials. They are potential candidates for stimuli-sensitive appliances, such as self-tightening and anchoring sutures/staples, surgical fasteners, orthopedic fixations and self-expandable vascular stents and implants, among others [1,2,3], with applicability in minimally invasive surgery. In this sense, these materials can be located in the body in a temporary and compressed/pre-deformed geometry through a small incision, to achieve their final/original and desired shape when heated to above the melting or glass transition temperature, causing less surgical stress. Additionally, biodegradable polymers with shape memory activation temperatures around body temperature are preferred for these types of implants, as they will cause less surgical stress and can degrade after a specific period of time, avoiding the need for their surgical removal. The activation temperature, commonly named T_trans_, can be the melting temperature, T_m_, of the crystalline phase of the polymer or the glass transition temperature, T_g_, depending on the nature of the polymer. Thus, in order to implement a shape memory polymeric system for applications in medicine, it is necessary to tailor its mechanical and thermal properties to fall into the desired range, which is not easily achievable.

Poly (lactic acid) (PLA) is a thermoplastic polymer that has found wide applications in the area of biomedicine in the past three decades, thanks to its good mechanical strength (pure PLLA, elastic modulus, E = 3–4 GPa; tensile strength = 50–70 MPa), degradability, biocompatibility and non-toxicity, as well as a good thermal shape memory performance around 60 °C and its T_g_ [4]. In spite of its strengths, PLA possesses well-known limitations such as its relatively poor processability, high stiffness and crystallinity, as well as its low elongation at break (ε = 2–10%) compared with other commercially available polymers of medical grade [4,5]. Moreover, from a shape memory point of view, its T_g_ is well above the body temperature, which limits its direct application and the exploitation of such an interesting ability when creating a medical device (PDLA, T_g_ = 50–54 °C; PLLA, T_g_ = 57–60 °C) [5]. In this sense, it is important to highlight that the SMP research of today should overcome the limitations of SMPs for tailored practical applications such as biomedical uses, especially when involving potential materials as PLA. Therefore, numerous strategies have been explored in order to tune the properties of PLA for specific applications, including copolymerization [6] and blending [7]. The first strategy is usually used to enhance the glassy state elasticity of PLA through the incorporation of an amorphous elastic phase with a low T_g_ to form a copolymer [8,9], which usually results in complex structures and a successful balance of shape memory performance and mechanical/thermal properties is not always reached [10,11]. Therefore, physical blending, either with miscible or immiscible components, is considered as a much more practical and economic way to modulate the properties of PLA [12,13,14]. Considering that, and taking advantage of the intrinsic compatibility among PLA and oligomers such as lactic acid oligomers (OLA), due to their similar chemical structure, we used OLA as a plasticizer for triggering the activation temperature (T_trans_) of the shape memory effect, as well as the thermal and mechanical properties of PLA. We have previously studied different PLA/OLA formulations and processed them by electrospinning, being able to control the T_g_ and the moduli (loss and storage) in the ranges of 21–60 °C and 30–91 MPa, respectively [15]. Therefore, we were able to tailor the T_g_ of the system to fall into the range of operating temperatures useful for medical applications (T_g_ = 40–45 °C) and, thus, allow the shape memory effect of the PLA to be activated by body heat or a temperature slightly above body heat for samples containing 20–30 wt.% of OLA.

When using a polymeric material in a medical field, bacterial infection usually needs to be considered as, statistically, it causes half of all hospital infections. More importantly, the biocompatibility of SMPs can be limited due to residues of microorganisms even prior to sterilization. Although shape memory polymers have been largely studied for creating parts of biomedical devices, the introduction of antibacterial activity into these polymeric systems remains an important task and has been mainly limited to non-degradable shape memory polyurethane materials. Furthermore, the limitation of microorganism growth on SMP systems must be addressed. Bionanocomposites represent an inspiring route for creating new and innovative medical materials, and silver nanoparticles (AgNPs) have been reported to bring improvements in the mechanical properties of dental materials [16] and, more interestingly, in their antibacterial activity [17], as well as their sustained release [18] and osteo-integration [19]. Therefore, in the present work, we have synthesized chitosan-mediated silver nanoparticles (AgCH-NPs) with the aim of incorporating them into a PLA–OLA SMP matrix at different weight ratios, to obtain appropriate formulations with antimicrobial activity for their use as thermoplastic medical materials. The microstructural, morphological and antibacterial properties of synthesized AgCH-NPs as well as obtained nanocomposites were studied in a previous work [20]. The obtained balanced mechanical properties (ductility and toughness), together with the bactericidal effect and the non-toxicity of the AgCH-NP synthesis method, led us to examine their potential application as functional smart materials of interest in biomedicine. Consequently, to that end, the present work explores, in detail, the thermal, dynamo-mechanical and shape memory properties, as well as the antifungal activity, of the developed nanocomposites. The resulting shape memory polymeric systems are expected to provide shape recovery of the permanent shape and, at the same time, wider biocidal activity, broadening the application of PLA in the field of medicine.

## 2. Materials and Methods 

### 2.1. Materials

Chitosan from shrimp shells (deacetylation degree > 75%) and silver nitrate (AgNO_3_) were purchased from Sigma-Aldrich (St. Quentin Fallavier, France). Sodium hydroxide and acetic acid were purchased from Fluka (Seelze, Germany). Lactic acid oligomer (OLA) (Glyplas OLA8, ester content > 99%, density 1.11 g/cm^3^, viscosity 22.5 mPa∙s, molecular weight 1100 g/mol) was a gift from Condensia Quimica SA (Barcelona, Spain) and polylactic acid (PLA3051D, 3% of D-lactic acid monomer, molecular weight 142 × 10^4^ g/mol, density 1.24 g/cm^3^) was provided by NatureWorks^®^ (Naarden, The Netherlands).

### 2.2. Processing of Shape Memory Plasticized PLA/OLA Nanocomposites

Chitosan-mediated silver nanoparticles (AgCH-NPs) were synthesized following a previously reported protocol [21,22], obtaining spherical nanoparticles with diameters about 8 nm [20]. Nanocomposites of PLA–OLA containing the synthesized AgCH-NPs were obtained at 180 °C with a rotation speed of 100 rpm, in a microextruder equipped with two twin conical co-rotating screws (Thermo Scientific, MiniLab Haake Rheomex CTW5, Karlsruhe, Germany, 7 cm^3^ capacity). Firstly, PLA was added to the MiniLab and after 2 min, when it had reached the melt state, OLA and AgCH-NPs were loaded and mixed for 1 more minute (total mixing/residence PLA time of 3 min). Obtained blends were compression molded at 180 °C in a hot press (Collin P-200-P, Maitenbeth, Germany); after 1 min, to ensure melting, materials were subjected to 5 MPa for 1 min and subsequently cooled down to room temperature while retaining the pressure for a further 1 min. All the materials were dried previous to their processing, and the amounts of polymer (PLA), plasticizer (OLA) and nanoparticles (AgCH-NPs) were calculated in order to obtain nanocomposites with approximately 0.5, 1 and 3 wt.% of AgCH-NPs with respect to the PLA amount.

### 2.3. Characterization Techniques

Viscoelastic measurements of the pure PLA–OLA matrix and nanocomposites were carried out in a DMA/SDTA861e Dynamic Mechanical analyzer (Mettler-Toledo, Greifensee, Switzerland). Dynamo-mechanical thermal analyses (DMTA) were carried out from −60 to 120 °C with isothermal steps of 5 °C, in the range of 0.1–1 Hz at 3 steps per decade (0.1, 0.5 and 1 Hz). Samples with 4 mm width and ~100 µm of average thickness were measured in tensile mode with 10 mm of effective length between clamps. Strain amplitude was kept constant at 15 µm. Analyses were performed at least thrice per sample and the average was taken as representative values. Storage and Loss moduli, and Tangent Delta were recorded as a function of temperature and time, and glass transition temperatures (T_gDMTA_) were calculated as the maximum in Tangent Delta peak. Shape memory properties were quantified in cyclic-thermomechanical tensile tests consisting on a heating-stretching-cooling protocol implemented in a Q800 dynamo mechanical analyzer (TA Instruments, New Castle, DE, USA). Each single cycle included programming the temporary shape and recovering the permanent shape, as follows: (1) programming step—a temporary fixed shape was created under strain-controlled conditions. The sample was first heated up and equilibrated at 45 °C (a useful temperature for biomedical applications) and stretched to a maximum strain of 50% by applying a force ramp of 0.2 MPa/min. Then, the sample was cooled down and equilibrated at 10 °C and maintained while the stress was released to zero at 0.5 MPa/min, to fix the temporary shape; (2) recovery step—once the stress was released, the sample was heated again up to 45 °C at 3 °C/min to recover the permanent shape. Then, a subsequent cycle was started and the protocol was repeated four times. Strain fixity ratio (R_f_) represents the ability to fix the mechanical deformation (ε_m_) applied in the programming step; strain recovery ratio (R_r_) represents the ability of the material to memorize/recover the permanent shape after the programming step. Both were quantified with the following equations [23,24,25]:

R_f_ (N)% = (ε_u_ (N)/(ε_m_ (N)) × 100 (1)

R_r_ (N)% = ((ε_m_ (N) − ε_f_ (N))/(ε_m_ (N) − ε_f_ (N − 1))) × 100 (2)

where ε_m_ is the maximum strain after cooling and before unloading the sample and ε_u_ is the fixed strain after unloading at 10 °C and in the N^th^ cycle during the programming step. ε_f_ is the residual strain of the sample after the recovery step in the N^th^ cycle. The shape memory temperature profiles were selected based on the results obtained from the DMTA analysis at 1 Hz, and taking into account the fact that potential active medical devices are recommended to be activated in the range of 40–55 °C, a close range to physiological temperatures, which is not harmful for body tissues and avoids premature activation at room temperature [1].

The antimicrobial properties of the nanocomposites were determined against *Candida parapsilosis* (*C. parapsilosis*, ATCC 22019) fungi, following the E2149-13a standard method of the American Society for Testing and Materials (ASTM) [26]. Briefly, each nanocomposite was placed into a sterile falcon tube, filled with 10 mL of the fungi suspension (ca. 10^5^ colony forming units (CFU)/mL) and shaken at 150 rpm at room temperature for 24 h. Fungi concentrations at time 0 and after 24 h were calculated by the plate count method after a 48-h incubation period. Falcon tubes containing only the inoculum and neat, plasticized PLA were also used as control experiments.

## 3. Results

### 3.1. Glass Transition Temperature, Activation Energy and Crystallinity of the Systems

**Figure 1:**
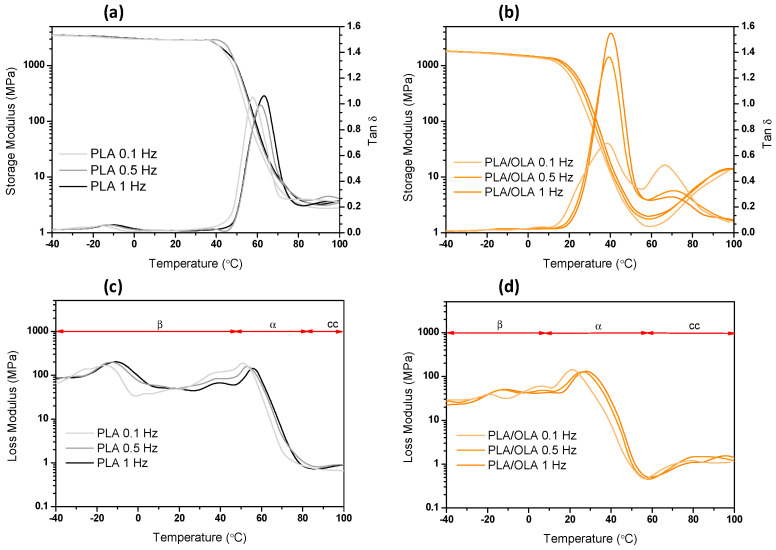
Temperature dependence of (**a**) poly (lactic acid) (PLA) and (**c**) PLA/lactic acid oligomer (OLA) storage modulus (E′) and loss tangent (tanδ) and (**b**) PLA and (**d**) PLA/OLA loss modulus (E″) at different frequencies (0.1, 1 and 3 Hz).

Regarding the shape memory effect in PLA-based materials, glass transition temperature can be used as the transition temperature of SMPs. Therefore, in order to better understand the shape memory behavior of the developed materials prior to studying their shape memory response, an insight into the effect of the addition of both OLA and AgCH-NPs in terms of the glass transition temperature is essential. In order to characterize the motions of pure PLA/OLA matrix on both crystalline and amorphous regions of the nanocomposites, dynamic mechanical measurements were carried out at multi-frequency temperature sweep mode at three different frequencies. The neat PLA matrix was also characterized for comparison purposes in order to better understand the transitions obtained with the incorporation of OLA. Figure 1 shows the temperature dependence of the storage modulus, E′, the loss tangent, tan δ, and the loss modulus, E″, for neat PLA and PLA/OLA at three different frequencies.

Figure 1d (PLA/OLA loss modulus) displays the existence of several transitions that fall into three main absorption regions from −30 to 10, 20 to 60 and 60 to 110 °C that have been labeled as β relaxation, α relaxation and cold crystallization region (cc), respectively. The sharp α peak, centered at around 40 °C in tan δ (Figure 1b), is accompanied by a sharp decrease in the storage modulus (E′) and corresponds to the segmental relaxation associated to the glass transition (micro-Brownian motions of long chain segments in the amorphous phase of the matrix). At temperatures below T_g_, secondary relaxation processes (β transitions) are appreciated, as broad shallow peaks result from the movements of localized groups of backbone atoms in the amorphous phase due to an increase in free volume with temperature. After T_g_ relaxation, a slight increase in E′ and E″ is accompanied with a transition peak in tan δ, which is attributed to cold crystallization processes. As occurs with PLA/OLA [27], in PLA three distinct regions can be differentiated. Apparently, the β transitions due to secondary relaxation processes seem not to be highly affected, while glass transition relaxation is clearly diminished, probably in association with the increase in free volume between chains due to the plasticizing effect of OLA. As expected, an increase in the frequency produces a shift to higher temperatures of the transitions, which is in line with results from the literature [28].

**Figure 2:**
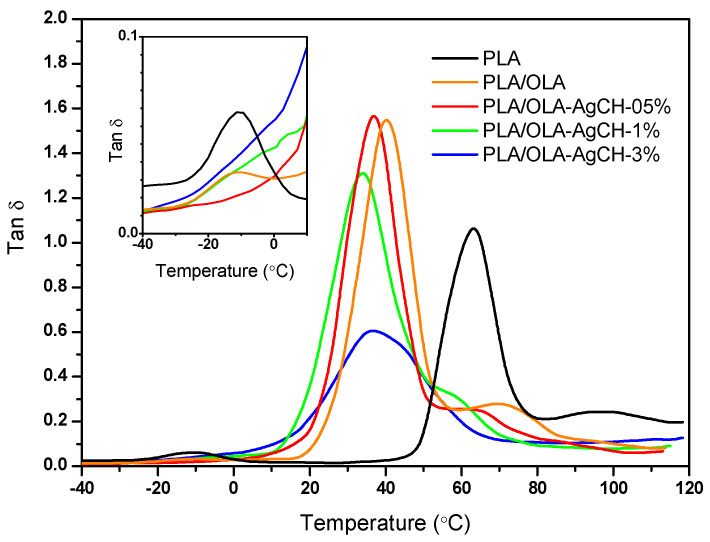
Temperature dependence of PLA, PLA/OLA and PLA/OLA-AgCH nanocomposite delta tangents (tan δ) at a frequency of 1 Hz.

Figure 2 shows the temperature dependence of tan δ for neat and plasticized PLA and for all the nanocomposites at a frequency of 1 Hz.

**Figure 3:**
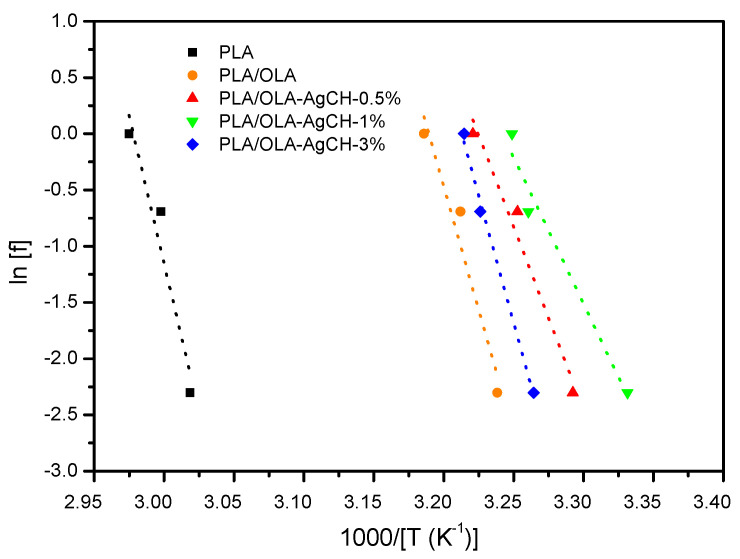
Plot of (1/T_g_) vs. ln*f* based on tan δ peaks for the different systems.

As can be seen in Figure 2**,** the α relaxation corresponding to the glass–rubber transition of samples shifts to a lower temperature with the incorporation of OLA into the PLA matrix. Additionally, the β-relaxation shoulder decreases with both the incorporation of OLA and increases in AgCH-NPs. We observe the same number of absorption peaks for all samples; therefore, it seems that, qualitatively, there are no dramatic changes in the relaxation processes between PLA and PLA/OLA and the nanocomposites at a frequency of 1 Hz. To explore this effect in more detail, we have calculated the characteristic relaxation time of the glass transition relaxation process using the maximum of the delta tangent peak (T_g_) for the three frequencies investigated by means of the following expression:

τ(T_α,β,γ_) ∝ (1/2πf) (3)

where τ is the characteristic relaxation time, T is the temperature at the maximum of the relaxation peak, and f is the experimental frequency. The dependence of the characteristic relaxation time on temperature, τ(T), is demonstrated in Figure 3. This dependence can be evaluated using the Arrhenius law, that is:

τ(T) ∝ exp(*E_a_*/(k_B_T)) (4)

where *E_a_* is the activation energy of the corresponding relaxation process. The values of *E_a_* for α relaxation process for pure PLA, plasticized PLA/OLA matrix and the nanocomposites together with the values of T_g_ at studied frequencies (0.1, 0.5 and 1 Hz) are depicted in Table 1. As previously observed in neat PLA and PLA/OLA, by increasing the test frequency, an increase in T_g_ occurs in nanocomposite samples.

Figure 3 shows the values of ln*f* and (1/T_g_) for T_g_, determined as the maximum in tan δ. The activation energy related to the glass transition is obtained by multiplying the slopes of the 1/T vs. ln*f* plot with the gas constant, R = 8.314 × 10^−3^ kJ mol^−1^ K^−1^. The *E_a_* values for the glass transition of the neat PLA and PLA/OLA systems are estimated as 435 and 365 kJ, respectively.

DMTA results evidenced lower glass transition values and *E_a_* for PLA/OLA and its nanocomposites in comparison with neat PLA. The addition of OLA shifts the T_g_ towards lower values, evidencing a plasticizing effect. Furthermore, it is noticed that the glass transition temperature decreases with the addition of AgCH-NP content up to 1 wt.% and then increases with a further increase in nanoparticles (3 wt.%) being closer to the neat PLA/OLA. This increase, with a high nanoparticle load, can be attributable to the confinement effects of polymer chains between nanoparticles, restricting chain mobility near the surface of AgCH-NPs [29,30]. The activation energy trend agrees with the tendency of T_g_; thus, the activation energy decreases for contents up to 1 wt.% of AgCH-NPs with respect to neat PLA/OLA, while reaching the highest value for 3 wt.% of AgCH-NP load. This fact reflects the fact that high AgCH-NP loads (3 wt.%) started to hinder the molecular motions of polymeric chains and the fact that higher *E_a_* is needed than for neat PLA/OLA.

Underlying thermal mechanisms in triggered semicrystalline SMPs involve the glass transition temperature (T_g_) and crystallinity degree. Therefore, as both properties can significantly affect the shape memory performance, it is also worth studying the extent of the effect that the addition of OLA and AgCH-NPs produces over the T_g_ and degree of crystallinity of the samples in detail. Table 2 summarizes the T_g_ values obtained from differential scanning calorimetry (DSC), as previously reported [20], and DMTA, as well as the degree of crystallinity. Both T_g_ values (from DMTA and DSC), along with numerical differences, follow the same trend. Similar observations have been reported widely in the literature and are usually related to the differences found in the fundamental working principle of each technique as well as in the sample scale size [31,32]. Regardless of those differences, as commented before for tan δ and taking neat PLA as reference, the addition of either OLA or AgCH-NP significantly lowers both T_gDSC_ and T_gDMTA_. As can be deduced from Table 2, the decrease in PLA glass transition is accompanied by an increase in melting enthalpy, suggesting that OLA promotes PLA crystal nucleation, as evidenced through the calculated degree of crystallinity. This is in line with observations done in other PLA plasticized systems and probably occurs due to the lowering of the interfacial surface energy of the plasticized molecules facilitating PLA crystal nucleation [33,34].

Regarding the nanocomposites, an important observation is that amounts of AgCH-NPs from 1 to 3 wt.% induced crystallinity into the PLA/OLA matrix while retaining lower T_gDMTA_ and *E_a_*. This, in principle, can be related to reduced entanglements/interactions occurring among nanoparticles and polymer chains into the amorphous domains, favoring their mobility and therefore reducing the T_g_ and *E_a_*, as previously reported in other nanocomposite systems [35].

**Figure 4:**
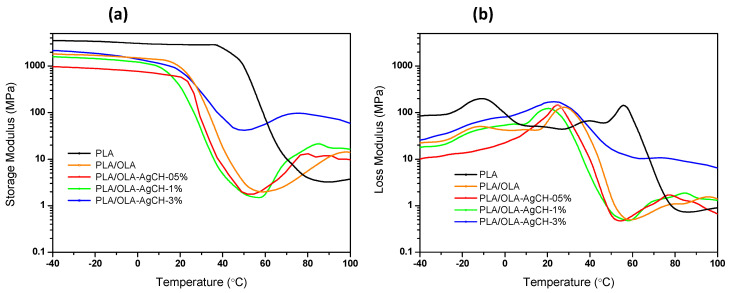
Evolution of (**a**) storage modulus (E′) and (**b**) loss modulus (E″) as a function of temperature.

Apart from tan δ, the storage modulus and loss modulus can give a good insight into the understanding of the shape memory performance. Figure 4 shows the evolution of the storage modulus (E′) and loss modulus (E″) as a function of temperature. As expected, the addition of OLA decreases the E′ and E″ values of the system at low temperatures. The loss modulus width broadens with the increase in AgCH-NP content at the same time that the maximum moves to lower temperatures in comparison to PLA/OLA samples. These results illustrate that molecular mobility is more easily activated for nanocomposites containing 0.5–1 wt.% of AgCH-NPs in agreement with calculated *E_a_*.

**Figure 5:**
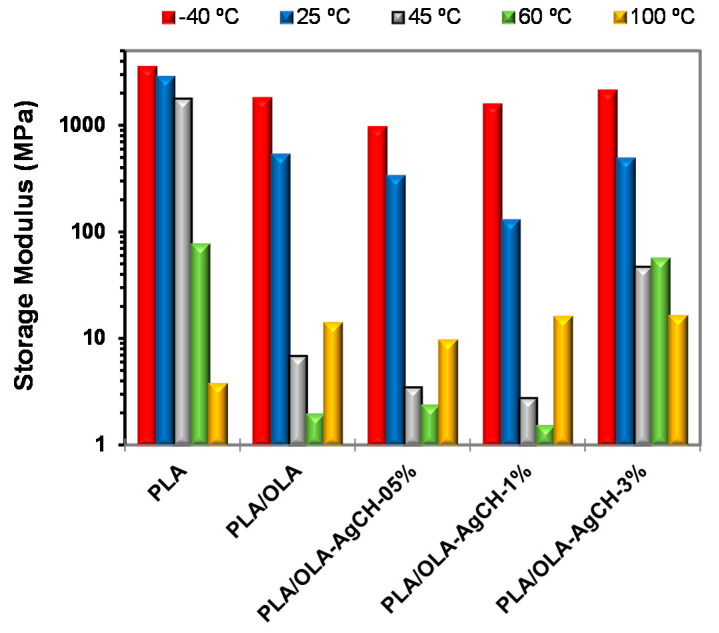
Storage Modulus, E′, at different temperatures.

As can be observed in Figure 5, in the glassy state (at −40 °C), PLA/OLA and its nanocomposites containing 1–3 wt.% of AgCH-NPs show similar E′ values, 1811 MPa, 1578 MPa and 2134 MPa for neat PLA/OLA, 1 and 3 wt.% for AgCH-NP nanocomposites, respectively, while, for a lower amount of filler (0.5 wt.%), a slight decrease in the modulus occurs (966 MPa), which can be related to the absence of crystallinity in this sample. At ambient temperature (25 °C), the stiffness starts to display a reduction as all the samples are close to glass transition temperature and, upon increasing the temperature to 45 °C, the stiffness displays a drastic reduction, reaching values of ~4 MPa for nanocomposites containing 0.5–1 wt.% of AgCH-NPs and ~7 MPa for neat PLA/OLA. Interestingly, the addition of 3 wt.% of AgCH-NPs leads to a substantially higher reinforcement of the E′ moduli compared to neat PLA/OLA. Moreover, this reinforcement effect is retained despite the increase in the temperature and the cold crystallization.

### 3.2. Shape Memory Properties; Thermal Activation

**Figure 6:**
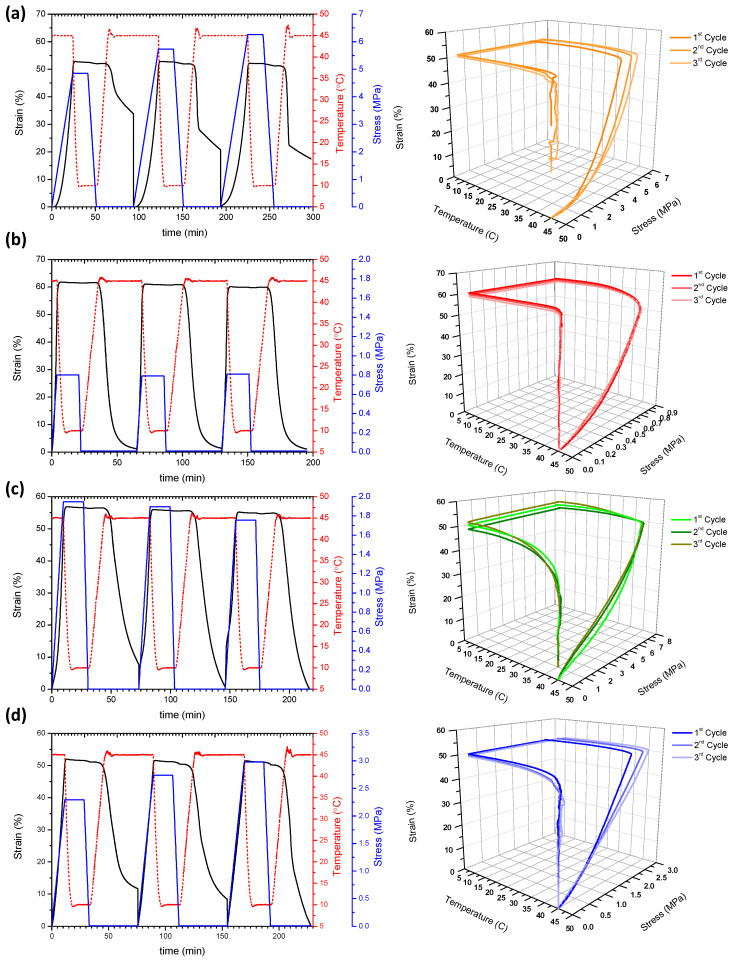
2D and 3D thermo-mechanical cycles performed at 45 °C for (**a**) neat PLA/OLA and its nanocomposites containing (**b**) 0.5 wt.% of AgCH-NPs, (**c**) 1 wt.% of AgCH-NPs and (**d**) 3 wt.% of AgCH-NPs.

As mentioned before, nowadays, PLA represents one of the most exceptional polymers in the biomedicine field for controlled drug delivery and tissue engineering. Nevertheless, in spite of its benefits, its inherent brittleness has been limiting its use. In this sense, the shape memory effect of neat PLA has been theorized by many research groups [36,37,38]; however, it was not observed due to its brittleness at room temperature. Therefore, in order to avoid the brittle fracture of the material, mainly polyurethanes (TPU) and poly (ε-caprolactone) polyester, have become common choices for blending or copolymerizing with PLA to create useful and flexible SMP [39,40]. Our research approach using lactic acid oligomer (OLA) as plasticizer ensures the compatibility of the blend and contributes to improving the ductility of the PLA, decreasing the glass transition to temperatures useful for biomedical applications. Thus, considering the results obtained in the DMTA analysis for the addition of AgCH-NPs, and to fulfill the requirements for further applications in biomedicine—in terms of facilitating the manipulation, storage and implantation of a possible device—a temperature of 45 °C has been selected for the activation of the shape recovery [1]. As previously explained in the experimental section, the shape memory properties were quantified through shape recovery (R_r_) and shape fixity (R_f_) ratios in three consecutive thermomechanical cycles in a DMTA. Temporary shape was programmed at 45 °C (T_high_ > T_gDMTA_ determined from the maximum in the tanδ curves) by elongating the sample until it reached 50% strain, then it was fixed at 10 °C (T_low_ ˂ T_gDMTA_) under constant stress. After removing the load, recovery was performed at 45 °C. Figure 6 shows the evolution of stress and strain as a function of time and temperature for neat PLA/OLA and its AgCH-NP nanocomposites. Table 3 collects the calculated numerical values for R_r_ and R_f_ as well as stress at maximum deformation.

**Figure 7:**
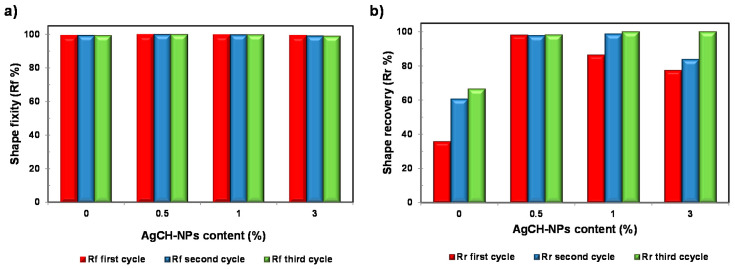
Shape fixity ratio (**a**) and shape recovery ratio (**b**) characteristic values for neat PLA/OLA and its nanocomposites containing 0.5, 1 and 3 wt.% of AgCH-NPs.

Under the conditions used, shape fixity ratios remained constant for all the samples and close to unity (R_f_ = 99–100%), without the appreciable influence of the AgCH-NP content or the number of cycles. Conversely, shape recovery ratios (R_r_) were higher for all the nanocomposites compared to neat PLA/OLA, which showed an incomplete shape recovery (lower than 50% for the first cycle). Figure 7 details the graphical evolution of shape memory parameters (R_f_ and R_r_) as a function of AgCH-NP content, complementing the information in Table 3. By incorporating AgCH-NPs into PLA/OLA, the recovery ratio in the first cycle improves, reaching the optimum at 0.5 wt.% of AgCH-NPs with an R_r_ of 98% and then, despite the better performance of the nanocomposites, a reduction in R_r_ to 86% and 77% was observed for samples with 1 and 3 wt.% of AgCH-NP content, respectively. According to DMTA results, these lower R_r_ values for the first cycle, with increasing AgCH-NP content, might be attributed to the enhancement of crystallinity and the more limited movement of amorphous polymer chains. Shape recovery is driven by the entropic stresses in the amorphous phase and, as shown in Figure 2, the magnitude of the tan δ transition decreases with increasing nanoparticle content, indicating that less mobile units are involved in the relaxation process and thus higher hysteresis occurs. With the increasing cycle number in the shape memory test, R_r_ shows an increasing trend, stabilizing near 100% after the second or third cycle for 1 and 3 wt.% of AgCH-NPs containing samples. Besides this training effect of R_r_ with cycles, strain recovery showed a strong dependence on nanoparticle content, whereas this dependence seems negligible for strain fixity performance.

The stress at maximum deformation (Table 3) is lower for all the nanocomposites compared to pure PLA/OLA. This fact is in accordance with the effect of the AgCH-NP addition over the mechanical properties of PLA/OLA matrices reported in our previous work [20].

**Figure 8:**
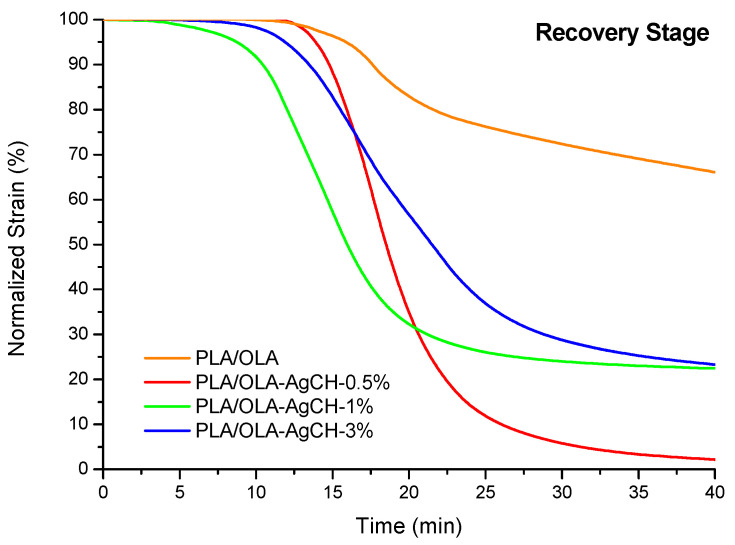
Normalized recovered strain with time for neat PLA/OLA and its nanocomposites containing 0.5, 1 and 3 wt.% of AgCH-NPs.

Figure 8 shows the evolution over time of the normalized strain during the recovery stage of the first cycle of the shape memory, for neat PLA/OLA and its nanocomposites at 45 °C. It could be seen that, for the same recovered strain (i.e., 80% of strain, thus 20% of strain recovered), all the nanocomposites presented faster recovery than the neat PLA/OLA, with the fastest being the nanocomposite containing 1 wt.% of AgCH-NPs. All the materials presented their T_g_ at lower temperatures than the set for recovery (45 °C) and, in spite of similar glass transitions to neat PLA/OLA and 3 wt.% of AgCH-NP samples, the recovery of the latter is faster. Thus, it seems that the presence of AgCH-NPs provides an extra force to the PLA/OLA matrix, helping the molecular chains to return to their original configuration.

These results demonstrate that the better overall performance of the nanocomposites might also be due to improved local heating and more efficient heat transfer during heating, which leads to less dissipation of energy, a faster response and higher shape recovery.

### 3.3. Antifungal Activity

In a previous work, these nanocomposites were tested against Gram-positive *Staphylococcus aureus* and Gram-negative *Escherichia coli* bacteria, showing that they exhibited antibacterial activities due to the dual combination action of silver ions and the cationic chitosan [20]. In this work, we extended our analysis of their antimicrobial behavior, testing them against *C. parasilopsis* fungi. Fungi present different membrane structures than bacteria; fungal walls are composed of chitin and polysaccharides, while bacteria are mainly made of peptidoglycans, lipoproteins and others. Therefore, the antibacterial activity of nanocomposites can be different than the antifungal effectivity.

The antifungal activity of nanocomposites was tested by the ASTM standard method [26]. Table 4 summarizes the antifungal activity represented as the percentage of fungi killed, which was calculated by the difference between the CFU after contact with control substrates (PLA/OLA and none), *A*, and CFU after contact with the polymeric nanocomposites, *B*, following the equation:

Percentage of fungi killed = (*A* − *B*)/*A* × 100 (5)

As can be seen, all nanocomposites exhibited significant antifungal activity. It is worth noting that the incorporation of only 0.5 wt.% of chitosan-mediated silver nanoparticles (AgCH-NPs) was able to kill 99% of fungi present in the tested suspension in contact with the film.

## 4. Conclusions

In this study, plasticized PLA-based SMP nanocomposites with tailored functionality were prepared with different amounts of chitosan-mediated silver nanoparticles, AgCH-NPs (0.5, 1 and 3 wt.%). Nanocomposites were obtained by melt-compounding in a twin-screw extruder, and were subsequently hot pressed. A comprehensive evaluation of the performance of the neat plasticized matrix as well as the nanocomposites was conducted by focusing on their dynamo-mechanical, shape memory and antimicrobial properties. To that end, their dynamic thermo-mechanical and shape memory behavior, as well as antifungal properties, were studied. The plasticizing effect of OLA notably lowers the T_g_ value of the neat PLA by at least 30 °C, according to DMTA measurements. In addition, a significant decrease also occurs in the storage and loss modulus at temperatures close to the physiological one of interest. PLA/OLA based nanocomposites showed a complex thermo-mechanical behavior, where the presence of the AgCH-NPs, especially high loads (3 wt.%), sufficiently affected the crystallinity and thermo-mechanical properties. Importantly, the addition of 0.5–1 wt.% of nanoparticles into the PLA/OLA matrix slightly reduces the glass transition temperature, while loads of 3 wt.% hinder the molecular motions of the chains, increasing the T_g_ to values closer to the neat material. Although an increase in the T_g_ and activation energy occurs in the nanocomposite with the highest load, those values are still below the ones of neat PLA/OLA. The presence of AgCH-NPs is responsible for the enhanced crystallinity, the lower glass transition and activation energies, as well as the reinforcement of the storage modulus in the rubbery region (45–100 °C) of the nanocomposites in comparison to the neat matrix. All these facts together are the driving forces for the better overall performance of the nanocomposites in the shape memory tests at the temperature of interest, resulting in higher and faster recovery ratios. In this sense, AgCH-NPs seem to provide an extra force to the PLA/OLA matrix to return the original configuration. In addition, the nanocomposites showed excellent antimicrobial effects, which, combined with the enhancement of their shape memory properties, give these materials great potential for performing as substrates with multi-functionality in the biomedical field.

## Figures and Tables

**Table 1:** Dynamo-mechanical thermal analyses (DMTA) results of T_g_.

Sample	Frequency [Hz]	^1^ T_g_ [°C]	^1^*E_a_* [kJ]
PLA	1	63	435
	0.5	60	
	0.1	58	
PLA/OLA	1	41	365
	0.5	38	
	0.1	36	
PLA/OLA-AgCH-0.5%	1	37	270
	0.5	34	
	0.1	31	
PLA/OLA-AgCH-1%	1	35	215
	0.5	34	
	0.1	27	
PLA/OLA-AgCH-3%	1	37	410
	0.5	37	
	0.1	33	

^1^ Standard errors (±): 1 °C for temperatures; 5 kJ for *E_a_*_._

**Table 2:** Thermal properties and crystallinity calculated from differential scanning calorimetry (DSC) scan for neat PLA and PLA/OLA and nanocomposites formulations containing AgCH-NPs.

Sample	^2^ T_gDMTA_ [°C]	^1,2^ T_gDSC_ [°C]	^1,2^ T_cc_ [°C]	ΔH_cc_ [J/g]	^1,2^ T_m_ [°C]	ΔH_m_ [J/g]	ΔH_Total_ [J/g]	^1^ X_c-DSC_ [%]
PLA	63	62	123	2	149	2	0	--
PLA/OLA	41	32	88	25	143	27	2	2.8
PLA/OLA AgCH0.5%	37	25	83	27	142	27	0	0.0
PLA/OLA AgCH1%	35	24	76	23	142	29	6	9.2
PLA/OLA AgCH3%	37	50	66	2	142	30	28	38.0

^1^ From reference [20]. ^2^ Standard errors (±): 1 °C for temperatures, 1 J/g in ΔH_m_ and 5% for **X_c_**.

**Table 3:** Strain recovery, strain fixity and stress at maximum strain values for each shape memory cycle.

Sample	Cycle [N°]	R_r_ (N) [%]	R_f_ (N) [%]	Stress at Max. Strain [MPa]
PLA/OLA	1	36	99	4.9
	2	61	99	5.6
	3	67	99	6.0
PLA/OLA-AgCH-0.5%	1	98	100	0.8
	2	98	100	0.8
	3	98	100	0.8
PLA/OLA-AgCH-1%	1	86	100	1.9
	2	99	100	1.9
	3	100	100	1.7
PLA/OLA-AgCH-3%	1	77	99	2.3
	2	84	99	2.7
	3	100	99	3.0

**Table 4:** Killing percentage for *C. parapsilosis* fungi by nanocomposite samples.

Sample	Killing Percentage [%]
PLA	-
PLA/OLA	-
PLA/OLA CH	90
PLA/OLA-AgCH-0.5%	99
PLA/OLA-AgCH-1%	99
PLA/OLA-AgCH-3%	99
